# Evaluating the Diagnostic Utility of Serum Laboratory Studies and Synovial Fluid Analysis in Identifying Periprosthetic Joint Infection in Metal Hip Revisions

**DOI:** 10.7759/cureus.70823

**Published:** 2024-10-04

**Authors:** Andrea H Johnson, Jane C Brennan, Justin J Turcotte, Paul J King

**Affiliations:** 1 Orthopedics, Anne Arundel Medical Center, Annapolis, USA; 2 Orthopedic Research, Anne Arundel Medical Center, Annapolis, USA; 3 Orthopedic and Surgical Research, Anne Arundel Medical Center, Annapolis, USA; 4 Orthopedic Surgery, Anne Arundel Medical Center, Annapolis, USA

**Keywords:** adverse reaction to metallic debris (armd), leukocyte count, metal-on-metal bearing, periprosthetic joint infection (pji), polymorphonuclear cell differential, revision surgery, synovial fluid analysis, total hip arthroplasty

## Abstract

Background

Metal-on-metal (MoM) bearing surfaces have been implicated as a cause of increased complication rates in total hip arthroplasty (THA), with local and systemic reactions identified. These reactions may cause abnormal laboratory results in common tests that are used to diagnose periprosthetic joint infection (PJI). The purpose of this study was to evaluate the utility of common laboratory studies in the diagnosis of PJI in patients undergoing revision THA with MoM bearings.

Methods

A retrospective case series of 81 patients undergoing THA revision of MoM bearings from January 2010 to May 2023 at a single institution was performed. Patient data was extracted from the electronic medical record. All patients were evaluated using the 2018 International Consensus Meeting (ICM) definition of PJI. Descriptive statistics were calculated for the clinical characteristics of all patients. Univariate analyses were performed to compare patients who met the ICM criteria for infection with those deemed indeterminate.

Results

Fifty-one (63.0%) patients were deemed not infected according to ICM criteria, 19 (23.5%) were deemed indeterminate, and 11 (13.6%) were deemed infected. Clinically, four patients had two or more positive cultures and were formally treated for PJI; all patients were deemed inconclusive, and the remaining seven patients deemed infected were clinically treated as not infected, and all remained infection-free. There were significant differences between patients deemed inconclusive and those deemed infected in synovial WBC count (17,539 vs. 90,196 cells/μL, p = 0.049) and synovial polymorphonuclear (PMN) neutrophils (38 vs. 79%, p = 0.002). No other significant differences in laboratory values or outcomes were noted between groups.

Conclusions

Patients undergoing revision THA with MoM bearings may be more likely to present with a clinical picture that is concerning for infection and may benefit from a more aggressive preoperative workup. The synovial PMN neutrophil percentage may help differentiate between hips that are infected and those that are not.

## Introduction

Metal-on-metal (MoM) bearing surfaces in total hip arthroplasty (THA) were popularized again at the beginning of the 21st century in recognition of the larger available head sizes and decreased wear rates benefiting younger and more active patients [1,2]. With increased usage of MoM constructs, there began to be concerns about local and systemic complications related to metal debris and the release of cobalt and chromium ions [3]. Surgeons began seeing increased complication rates associated with MoM-bearing surfaces, including adverse local tissue reaction (ALTR), pseudotumor, aseptic lymphocytic vasculitis-associated lesions, and serum metallosis [4,5]. An increased rate of revision of MoM THA for infection has also been noted [2]. Due to all of these factors, MoM bearings have fallen out of favor, and usage of these bearings has decreased precipitously since 2007 [6].

The standard laboratory tests used to diagnose periprosthetic joint infection (PJI) include serum erythrocyte sedimentation rate (ESR), serum CRP, synovial WBC count, and polymorphonuclear (PMN) cell differential may have limited utility in MoM THA due to the local and systemic reactions that have been described [7]. A number of studies have evaluated the utility of each of these tests in the setting of revision THA of an MoM bearing, although there is not a consensus on the accuracy or utility of the tests [4,7-9]. PJI is the cause for approximately 12-15% of all revision THAs performed in the United States currently, and the total number of revisions for infection is likely to increase as the rate of revision THA increases [10,11]. Given the significant morbidity, mortality, and cost to both the patient and the healthcare system as a whole that is associated with PJI, it is important to identify parameters to accurately diagnose or exclude PJI in patients undergoing revision THA with MoM bearings.

The purpose of this study was to evaluate the utility of laboratory studies of patients undergoing revision THA of an MoM bearing in order to determine whether they met the criteria for a PJI diagnosis.

## Materials and methods

This study was deemed exempt as a retrospective review of existing medical records by the Western Institutional Review Board.

International Consensus Meeting (ICM) infection criteria

All patients were evaluated using the 2018 ICM definition of PJI and categorized as noninfected, indeterminate, or infected [12]. At least one of the following was a major criterion: two positive cultures of the same organism using the standard culture method or sinus tract with evidence of communication to the joint or visualization of the prosthesis. Major criteria constituted an immediate classification as infected. Minor criteria were assigned a point value, and a combined score of <3 was not infected, 3-5 were inconclusive, and 6 or more were infected. Table 1 outlines the minor criteria in detail.

**Table 1:** 2018 ICM minor criteria for diagnosing PJI A combined score of ≥6 indicates an infected joint; a combined score of 3-5 is inconclusive; and a combined score of <3 is not infected. ESR, erythrocyte sedimentation rate; ICM, International Consensus Meeting; PJI, periprosthetic joint infection; PMN, polymorphonuclear

Minor criteria	Acute infection	Chronic infection	Score
Serum CRP (mg/dL)	10	1	2
or			
D-Dimer (µg/L)	Unknown	860	
Elevated serum ESR (mm/hr)	No role	30	1
Elevated synovial WBC (cells/µL)	10,000	3,000	3
or			
Leukocyte esterase	++	++	
or			
Positive alpha defensin (signal/cutoff)	1	1	
Elevated synovial PMN (%)	90	70	2
Single positive culture			2
Positive histology			3
Positive intraoperative purulence			3

For the purpose of this study, the last serum CRP and ESR prior to revision THA was recorded if multiple results were available. Visualization of a sinus tract was documented in preoperative notes. Intraoperative purulence was recorded in the narrative operative note. Synovial fluid, fluid and tissue cultures, and histology were performed intraoperatively as indicated by the operating surgeon’s protocol.

Study population

A retrospective case series of 81 patients who had a revision of a MoM THA from January 2010 to May 2023 from a single institution was performed. Demographics, preoperative characteristics, laboratory results, operative findings, and postoperative findings were all extracted from the electronic medical record via manual chart review. Table 2 outlines the demographic characteristics of the population.

**Table 2:** Clinical characteristics Data are presented as n (%).

Characteristic	All patients (n = 81)
Age, years	67.1 ± 8.3
Women	43 (53.1)
BMI, kg/m²	30.0 ± 6.3
Time to revision, years	9.3 ± 4.3
Revision approach: direct anterior	16 (19.8)

Surgeries were performed by one of seven board-certified orthopedic surgeons who are fellowship-trained in joint arthroplasty. Patients who did not have at least one of the following lab values were excluded: preoperative serum CRP, preoperative ESR, synovial WBC, or synovial PMN neutrophils.

Table 3 identifies the primary mechanism of failure of the primary THA identified preoperatively. The most common reason for the revision was an adverse reaction to metallic debris (metallosis), accounting for 58 (71.6%) patients.

**Table 3:** Mechanism of failure for primary THA identified preoperatively One patient with a dislocation also had fibrous ingrowth of the acetabular component, while two patients with infections also exhibited metallosis. Data are presented as n (%). THA, total hip arthroplasty

Mechanism of failure	All patients (n = 81)
Recurrent dislocation	4 (4.9)
Infection	4 (4.9)
Fibrous ingrowth acetabular component	13 (16.0)
Metallosis	58 (71.6)
Periprosthetic fracture	2 (2.5)

Outcomes

The primary outcome of interest was the comparison of diagnostic values between the patients categorized as inconclusive versus infected by the ICM criteria. Secondary outcomes included the overall rate of PJI in this population and reasons for subsequent re-revision.

Data analysis

Descriptive statistics were calculated for the clinical characteristics of all patients. Patients were scored on their lab values and assigned an ICM infection classification. Chi-square and Kruskal-Wallis tests were conducted to determine differences in ICM criteria, intraoperative characteristics, and outcomes by ICM infection classification. All statistical analyses were performed using R Studio (Version 4.2.2 © 2009-2023 RStudio, PBC, Boston, MA, USA). Statistical significance was assessed at p < 0.05.

## Results

Fifty-one patients were deemed noninfected when scored according to the ICM criteria (Table 4).

**Table 4:** Laboratory and histology findings for patients identified as noninfected per ICM criteria Data are presented as n (%) unless otherwise indicated. ESR, erythrocyte sedimentation rate; ICM, International Consensus Meeting; PMN, polymorphonuclear

Criteria	Result (avg-range)	Percentage of patients (total N = 51) scoring points per ICM criteria
2+ positive cultures	-	0 (0)
Serum CRP (mg/dL)	0.58 (0.04-2.98)	5 (9.8)
Serum ESR (mm/hr)	11.8 (1-44)	3 (5.9)
Synovial WBC (cells/µL)	827.88 (9-2,938)	0 (0)
Synovial PMN (%)	53.79 (3-98)	7 (13.7)
Histology	-	0 (0)
Purulence		0 (0)
Single positive culture	-	0 (0)

Five patients had an elevated preoperative serum CRP, three patients had an elevated preoperative serum ESR, and seven patients had an elevated synovial PMN% intraoperatively. Table 5 compares the differences between the 19 patients deemed inconclusive for PJI according to the ICM criteria and the 11 patients who were deemed infected.

**Table 5:** Comparison of quantitative measures (per ICM criteria) between inconclusive and infected patients Data are presented as n (%). ESR, erythrocyte sedimentation rate; ICM, International Consensus Meeting; PMN, polymorphonuclear

Criteria	Inconclusive (n = 19)	Infected (n = 11)
2+ positive cultures	0 (0)	4 (36.4)
Elevated serum CRP	4 (21.1)	9 (81.8)
Elevated serum ESR	4 (21.1)	9 (81.8)
Elevated synovial WBC	12 (63.2)	10 (90.9)
Elevated synovial PMN	2 (10.5)	8 (72.7)
Positive histology	2 (10.5)	5 (45.5)
Purulence	0 (0)	4 (36.4)
Single positive culture	1 (5.3)	1 (9.1)

A higher percentage of infected patients had an elevated preoperative serum CRP and ESR and a higher synovial WBC count and PMN%. Of the 11 patients deemed infected, four had two or more positive intraoperative cultures of the same organism. One patient in each category had a single positive culture, both of which were deemed contaminants after further examination. All patients in the ICM inconclusive category were treated during and after revision as noninfected, with no patients undergoing extended antibiotic therapy or formal PJI revision surgery. Of the patients in the ICM-infected category, four were identified as having PJI prior to revision and were treated with a formal one- or two-stage revision and a minimum of six weeks of postoperative intravenous antibiotics. One required multiple additional revision surgeries to clear the infection but is ultimately infection free, and one had re-revision to reimplant a THA. The other seven patients in that category were treated as noninfected and had no extended antibiotic therapy or formal PJI revision surgery.

Table 6 compares the laboratory values of patients who were deemed inconclusive with those infected according to ICM criteria.

**Table 6:** Mean value of diagnostic values between inconclusive and infected hips Data are presented as mean ± SD, with p-values less than 0.05 indicated in bold. The analysis was conducted using an independent samples t-test. ESR, erythrocyte sedimentation rate; ICM, International Consensus Meeting; PMN, polymorphonuclear

Criteria	Reference range per ICM criteria	ICM inconclusive (n = 19)	ICM infected (n = 11)	p-value
Serum CRP (mg/dL)	1	1.1 ± 1.4	4.9 ± 6.9	0.125
Serum ESR (mm/hr)	30	29.4 ± 38.3	57.2 ± 28.8	0.067
Synovial WBC (cells/µL)	3,000	17,539.4 ± 36,308.2	90,196.3 ± 98,502.4	0.049
Synovial PMN (%)	70	38.2 ± 30.2	78.7 ± 25.4	0.002
Serum cobalt (ng/mL)	N/A	33.4 ± 41.2	31.6 ± 42.8	0.921
Serum chromium (ng/mL)	N/A	18.0 ± 24.0	24.4 ± 45.6	0.701

Patients who were deemed infected had a significantly higher synovial WBC count (17,539 vs. 90,196, p = 0.049) and a higher PMN% (38.2 vs. 78.7%, p = 0.002). There were no significant differences in preoperative serum CRP, ESR, cobalt, or chromium levels. Every patient in the ICM inconclusive or infected category had a synovial WBC above 3,000.

Table 7 compares the outcomes between patients in each ICM category.

**Table 7:** Outcomes by ICM classification Data presented as n (%) or mean ± SD. The analyses were performed using either the chi-squared test or independent samples t-test. ICM, International Consensus Meeting

Outcome	Not infected (n = 51)	Inconclusive (n = 19)	Infected (n = 11)	p-value
Re-revised	6 (11.8)	0 (0)	2 (18.2)	0.208
Reason for re-revision				0.345
Hematoma/wound healing	1 (2.0)	0 (0)	0 (0)	
Infection	1 (2.0)	0 (0)	1 (9.1)	
Mechanical failure	2 (3.9)	0 (0)	0 (0)	
Metallosis	1 (2.0)	0 (0)	0 (0)	
Pain/other	1 (2.0)	0 (0)	1 (9.1)	
Follow-up time, days	1,161.2 ± 976.3	1,079.8 ± 1,196.5	669.2 ± 700.4	0.982

There were no significant differences in the number of patients re-revised, the reasons for re-revision, or in follow-up duration. All patients in the ICM inconclusive category remained infection-free at their final follow-up. The seven patients in the ICM infected category that were treated as non-infected also remained infection-free at the final follow-up. One patient in the noninfected category did present with a PJI approximately 16 months after initial revision surgery, but it appeared to be unrelated to the initial revision rather than a missed diagnosis.

## Discussion

PJI is a devastating complication of THA and is associated with significant morbidity, mortality, and economic cost [13-16]. Diagnosing PJI continues to be a challenge, with a number of factors contributing to the diagnosis, including clinical exam findings, serum and synovial laboratory studies, fluid and tissue cultures, histological exams, and intraoperative findings [12]. Revisions of THA with MoM bearings complicate the picture even further as many of these patients have local and systemic reactions to metallic debris, which can mimic findings consistent with PJI [7]. At the same time, some studies have identified a higher rate of PJI in patients who have had a primary THA with MoM bearing [2,17-19]. In this study, nearly 14% of patients met the ICM criteria for infection; however, only four of these patients had multiple positive cultures during revision. This highlights the challenges surgeons face in accurately diagnosing and treating these patients. Next-generation genome sequencing technology may possibly provide earlier and more sensitive and specific detection of PJI and may be quite useful for this specific patient population [20,21].

Serum labs, including ESR and CRP, are some of the most important variables in the diagnosis of PJI [12]. These labs are a cost-effective way to diagnose a PJI preoperatively, requiring only a blood draw rather than a more invasive hip aspiration [22,23]. In the presence of a MoM bearing THA, the utility of serum labs has been more mixed, with some studies raising the concern of false positive results. In a study by Wyles et al., serum ESR and CRP were found to have poor diagnostic predictability for PJI [4]. In contrast, studies by Yi et al. and Kwon et al. found that these laboratory tests are useful in ruling out PJI as an initial screening in THA with MoM bearings [7,8]. In the current study, there was no significant difference in preoperative ESR and CRP between patients who were inconclusive for PJI according to the ICM criteria and patients who were infected.

Synovial WBC count is one of the most relied-upon tests for diagnosing PJI, in part because of the ease and speed of obtaining the results [24,25]. Multiple studies have shown high sensitivity when using synovial WBC to diagnose PJI [26-28]; there is some concern that relying on automated WBC counts may limit the utility of this test [24]. In the presence of MoM bearings, it is questionable whether synovial WBC count remains a sensitive test for diagnosing PJI due to metal debris and local tissue reactions that can mimic infection [4,7]. In a recent study by Bäcker et al., patients with MoM THA bearings had a significantly higher synovial WBC count than patients with ceramic on polyethylene bearings [29]. Wyles et al. found that the utility of synovial WBC was unclear in the presence of ALTR, although Kwon et al. found that synovial WBC was a good marker for diagnosing PJI [4,8]. In the current study, patients who were inconclusive for PJI according to the ICM criteria had a significantly lower synovial WBC count when compared to patients who were positive for infection; however, all patients in the inconclusive group had a synovial WBC count above the 3,000 threshold.

Along with synovial WBC count, differentials, particularly PMN, are very useful and an accurate measure in the diagnosis of PJI [4,25]. In a meta-analysis by Lee et al., synovial PMN% was found to have high sensitivity and specificity for diagnosing PJI [27]. While the overall WBC count does not perform consistently well in the presence of MoM bearings, the PMN seems to have greater diagnostic accuracy in these cases. Wyles et al. found that synovial PMN was a highly accurate indicator of PJI during revision THA in patients with MoM bearings, even as total synovial WBC count performed less well [4]. While metal debris and other foreign material from ALTR can obscure the differential in similar ways to the WBC count, it appears to be somewhat less affected overall [7]. Bäcker et al. found that while total synovial WBC count was elevated in MoM hips when compared with non-MoM hips undergoing aseptic revision, PMN% was not significantly different between these groups and was significantly lower than the PMN% in patients undergoing revision for PJI [29]. In the current study, there was a significant difference in PMN% between patients who were inconclusive for PJI according to the ICM criteria and patients who were infected.

The results of this study need to be considered in light of its potential limitations. It is a retrospective study from a single institution, and the results may not be comparable to the wider patient population. The ICM criteria were also applied after treatment had already taken place and did not necessarily reflect how the patients were actually treated (as infected or non-infected). Only four patients in this sample were actually treated as having a PJI, even though 11 patients met the ICM criteria for PJI. A significant number of patients also had serum metallosis, which has an unclear influence on the other lab values. The overall study size is small, and every patient that met the inclusion criteria was used; due to this, a power analysis was not performed. In addition, not every patient had every lab value available, so it may not have been categorized correctly. PJI remains a very difficult complication to diagnose and manage and requires looking at many different aspects, including laboratory values, histopathology, physical exam findings, and intraoperative findings.

## Conclusions

Patients undergoing revision THA after primary THA with MoM bearings may be more likely to present with a clinical picture that is concerning for infection, in the form of elevated inflammatory markers and/or elevated synovial cell counts. Systemic and local reactions to metallic debris are likely to contribute to this effect. These patients may benefit from a more aggressive preoperative workup, including preoperative joint aspiration for cell count and culture. Synovial PMN neutrophil percentage may help differentiate between hips that are infected and those that are not. Additional studies, ideally prospective studies, should be done to clarify appropriate diagnostic parameters in this population.
